# Mapping the Network Structure of Non-Suicidal Self-Injury: The Role of Emotional and Interpersonal Vulnerability and Attachment in Spanish Adolescents

**DOI:** 10.3390/ejihpe16070088

**Published:** 2026-06-25

**Authors:** Sandra Pérez-Rodríguez, Blanca Gallego-Hernández de Tejada, María José Beneyto-Arrojo, Xavier Sanz-Sendra

**Affiliations:** 1 Department of Personality, Assessment and Psychological Treatments, Universidad de Valencia, 46010 Valencia, Spain; sandra.perez@uv.es; 2 Departamento de Personalidad, Evaluación e Intervención Terapéutica, Universidad Católica de Valencia San Vicente Mártir, 46100 Valencia, Spain; blancagallego64@gmail.com (B.G.-H.d.T.); mariajose.beneyto@ucv.es (M.J.B.-A.); 3 Departamento de Psicología Básica, Neuropsicología y Social, Universidad Católica de Valencia San Vicente Mártir, 46100 Valencia, Spain

**Keywords:** non-suicidal self-injury, adolescence, emotion dysregulation, hopelessness, attachment, network analysis

## Abstract

Background: Non-suicidal self-injury (NSSI) is highly prevalent during adolescence and is associated with a range of emotional, cognitive, and interpersonal vulnerabilities. Although prior research has identified key correlates such as emotion dysregulation, hopelessness, interpersonal distress, and attachment insecurity, these factors have largely been examined in isolation, limiting understanding of how they jointly contribute to NSSI. Methods: The present study examined the network structure of NSSI and associated vulnerability processes in a community sample of 2067 Spanish adolescents (M age = 14.62, SD = 1.80). A regularized partial correlation network (EBICglasso) was estimated, including NSSI frequency and functions, emotion dysregulation, hopelessness, perceived burdensomeness, thwarted belongingness, and attachment representations. Centrality and network stability were evaluated using standard indices and bootstrapping procedures. Results: The network revealed a differentiated structure of associations. Perceived burdensomeness and intrapersonal NSSI functions emerged as the most influential nodes, whereas emotion dysregulation occupied a key bridging position connecting attachment-related experiences, interpersonal vulnerability, and NSSI processes. In contrast, NSSI frequency and interpersonal functions showed a more peripheral role. Attachment security was negatively associated with core risk variables, consistent with a protective role within the network. Conclusions: Findings suggest that NSSI in adolescence is embedded within a system of interacting emotional and interpersonal processes, structured around the functional meaning of the behavior and key interpersonal appraisals. Emotion dysregulation emerged as a highly connected node linking multiple domains, while attachment was associated with several key variables within the network. These findings suggest potential targets for early identification and intervention, particularly focusing on emotion regulation, perceived burdensomeness, and intrapersonal functions of NSSI.

## 1. Introduction

Non-suicidal self-injury (NSSI) refers to the intentional damage of one’s own body tissue in the absence of suicidal intent and outside socially sanctioned practices (International Society for the Study of Self-Injury, 2018). In adolescence, NSSI encompasses a heterogeneous set of behaviors, including cutting, burning, or hitting oneself, and frequently co-occurs with emotional, cognitive, and interpersonal difficulties (Klonsky & Glenn, 2009). Meta-analytic evidence indicates that NSSI is relatively prevalent during this developmental period, with global lifetime estimates ranging from 20% to 22% (Lim et al., 2019; Xiao et al., 2022), although prevalence varies widely depending on definitional and methodological criteria, and is substantially lower when DSM-5 diagnostic thresholds are applied (Dierickx et al., 2023; Esposito et al., 2023; Farkas et al., 2024; Swannell et al., 2014). This heterogeneity suggests that NSSI does not emerge in isolation but is embedded within a constellation of interrelated emotional and interpersonal processes that may vary across individuals and contexts (Tonta et al., 2023). In Spain, lifetime prevalence has been estimated at 24.6%, with a substantial proportion of adolescents engaging in severe methods or meeting DSM-5 criteria, underscoring the clinical relevance of examining NSSI within a broader network of associated risk factors (Perez et al., 2021).

Although NSSI is conceptually distinct from suicidal behavior, substantial evidence indicates a close association between the two. Meta-analytic studies show that a history of NSSI is linked to elevated risk of subsequent suicidal ideation, attempts, and mortality (Ribeiro et al., 2016; Victor & Klonsky, 2014). This association highlights the need to clarify the mechanisms underlying NSSI in order to inform the development of effective prevention and intervention strategies (Poudel et al., 2022; Turner et al., 2013, 2019). In this line of research, the Interpersonal Theory of Suicide (Joiner, 2005) identifies thwarted belongingness and perceived burdensomeness as central interpersonal contributors to suicidal desire (Joiner, 2005; Van Orden et al., 2010). Thwarted belongingness reflects unmet needs for social connection, whereas perceived burdensomeness refers to the belief that one’s existence is a burden on others (Van Orden et al., 2010). Within this framework, suicidal behavior is expected to emerge when suicidal desire co-occurs with acquired capability, which may develop through repeated exposure to pain, including NSSI.

Empirical evidence supports these associations. Thwarted belongingness has been linked to higher NSSI frequency (Assavedo & Anestis, 2016). In Spanish adolescent samples, both thwarted belongingness and perceived burdensomeness have been associated with lifetime NSSI (Marco et al., 2021), although more recent findings suggest associations may be specific to perceived burdensomeness (Perez Rodríguez et al., 2025).

On the other hand, hopelessness is conceptualized as a cognitive distortion characterized by diminished perceived control over the future and persistent negative expectations (Beck et al., 1974). It is a transdiagnostic construct implicated across multiple mental disorders (Beck & Steer, 1988). From an experiential perspective, hopelessness reflects a state of helplessness that undermines energy, hope, and future aspirations, particularly in contexts of limited social support (Sukma & Puspitasari, 2023). Adolescence represents a period of heightened vulnerability for the emergence of hopelessness (Sukma & Puspitasari, 2023).

Empirical evidence identifies hopelessness as a central factor in NSSI. The meta-analysis by Fox et al. (2015) highlights hopelessness as one of the most robust predictors of NSSI and suicide risk. Adolescents engaging in self-injury report significantly higher levels of hopelessness than those without NSSI (Gallego-Hernández de Tejada, 2019). Available evidence suggests no significant differences in hopelessness levels between adolescents who engage exclusively in NSSI and those who self-injure and report suicide attempts (Brausch & Gutierrez, 2010; Dhingra et al., 2016; Poudel et al., 2022). This pattern underscores the clinical relevance of early NSSI prevention as a key strategy to reduce subsequent suicidal ideation and attempts.

Emotion dysregulation refers to difficulties in monitoring, modulating, and expressing emotional responses in ways that are adaptive to situational demands. It involves the absence of regulatory efforts, the ineffective implementation of strategies, or the use of maladaptive responses that fail to reduce emotional distress (Gross et al., 1998; Wolff et al., 2019). These processes may operate consciously or unconsciously and can affect the timing, intensity, and duration of emotional experiences (Gross et al., 1998). Meta-analytic findings indicate a robust association between NSSI and difficulties regulating negative affect and emotional reactivity, with no moderation by age, gender, or sample type (Wolff et al., 2019). Although earlier evidence suggested more modest effects (Fox et al., 2015), subsequent studies have provided converging support for emotion dysregulation as a significant risk factor for the onset of NSSI (Hasking et al., 2017; Kiekens & Claes, 2020; Muehlenkamp & Tillotson, 2023; Thomas & Bonnaire, 2025). Importantly, recent evidence suggests that emotion dysregulation is also associated with higher levels of hopelessness, indicating that persistent difficulties in regulating emotional states may contribute to the development of negative future-oriented cognitions (Faura-Garcia et al., 2024). Converging findings further suggest that NSSI frequently functions as a maladaptive strategy to manage overwhelming emotional distress when regulatory capacities are compromised (Sanz-Sendra et al., 2024; Wang et al., 2024).

Attachment is conceptualized as an innate behavioral system through which individuals form enduring emotional bonds with caregivers, shaping emotional security across development (Bowlby, 1973). Early caregiving experiences give rise to internal working models that organize expectations about the self and others, as well as emotion regulation and behavior (Bowlby, 1973; Bretherton & Munholland, 2008). Sensitive caregiving fosters secure attachment and adaptive regulation, whereas neglectful or frightening care increases the likelihood of insecure or disorganized patterns (Ainsworth et al., 1978; Cassidy et al., 2018; Main & Solomon, 1986). Attachment insecurity has been consistently linked to greater vulnerability to NSSI in adolescence, partly through emotion dysregulation and relational difficulties (Chen et al., 2023; Gander et al., 2021). Across clinical and community studies, attachment insecurity has been consistently associated with recurrent or more severe self-injurious behaviors and poorer regulatory and problem-solving functioning, whereas attachment security has been related to more adaptive coping and indicators of recovery following NSSI (Glazebrook et al., 2015; Pallini et al., 2020; Woo et al., 2022).

Taken together, the existing literature suggests a consistent and interrelated pattern of associations among attachment, emotion dysregulation, hopelessness, perceived burdensomeness, thwarted belongingness, and NSSI during adolescence. Empirical studies have independently linked insecure and disorganized attachment with greater emotion dysregulation (Ozeren, 2022; Tatnell et al., 2018), elevated hopelessness (Ringer et al., 2014), and adverse interpersonal appraisals, including thwarted belongingness and perceived burdensomeness (Lev-Ari & Levi-Belz, 2019; Venta et al., 2014). In parallel, converging evidence from meta-analytic and empirical studies consistently identifies emotion dysregulation and hopelessness as key correlates of NSSI (Fox et al., 2015; Wolff et al., 2019; Gallego-Hernández de Tejada, 2019), while interpersonal factors derived from Joiner’s framework have also been linked to self-injurious behaviors in adolescent samples (Assavedo & Anestis, 2016; Marco et al., 2021; Perez Rodríguez et al., 2025).

Importantly, despite this convergence, these constructs have predominantly been examined in isolation or through unidirectional models, even though accumulating evidence indicates that they co-occur and may dynamically interact and reinforce one another over time. This convergence supports the conceptualization of NSSI as emerging from a network of interconnected emotional and interpersonal processes, rather than from a single underlying mechanism. Accordingly, a network approach provides a theoretically and methodologically appropriate framework to examine the structure of associations among these variables and to identify the most central and interconnected components within adolescent NSSI (Borsboom, 2017; Borsboom & Cramer, 2013; Epskamp et al., 2018; Fried et al., 2017).

Building on this framework, the present study aims to advance understanding of non-suicidal self-injury during adolescence by examining the network structure of key emotional, cognitive, and interpersonal vulnerability processes. Specifically, the study models the pattern of conditional associations among NSSI frequency and functions, emotion dysregulation, interpersonal needs (perceived burdensomeness and thwarted belongingness), hopelessness, and attachment representations. Using network analysis, the study seeks to identify the most central and influential nodes within this system, as well as the pattern of interconnections among variables, without imposing a priori assumptions about causal direction. By situating these processes within a developmental context, this approach aims to provide a more comprehensive account of how emotional dysregulation, interpersonal vulnerability, and attachment-related experiences are linked to NSSI in adolescence within a broader system of emotional and interpersonal processes.

## 2. Method

### 2.1. Participants

The sample consisted of 2067 adolescents attending secondary schools in Spain. Participants were recruited between February and March 2022 through collaboration with a nationwide network of charter schools. Although schools from nine autonomous communities were invited to participate, five institutions ultimately agreed—three in the Community of Madrid, one in the Basque Country, and one in the Valencian Community. Accordingly, the sampling strategy was non-probabilistic and based on convenience. Despite this limitation, the sample included adolescents from central, northern, and eastern regions of Spain, providing a degree of geographical diversity.

Most participants were Spanish nationals (98.6%, *n* = 2027). A small proportion of students (1.4%) were born outside Spain, representing countries from Europe, the Americas, Africa, and Asia.

The sample showed an approximately equal sex distribution, comprising 1072 boys (51.9%) and 995 girls (48.1%). Participants’ ages ranged from 11 to 19 years (M = 14.62, SD = 1.80). Most adolescents were in early to mid-adolescence, with the highest proportions observed at ages 12 (14.8%), 13 (17.2%), 14 (16.9%), 15 (15.9%), and 16 (16.3%). In contrast, representation at the boundaries of the age distribution was minimal, with only two participants aged 11 (0.1%) and fourteen aged 19 (0.7%). With respect to regional distribution, most adolescents were recruited in the Community of Madrid (64.6%, *n* = 1335), followed by the Basque Country (22.6%, *n* = 467) and the Valencian Community (12.8%, *n* = 265).

### 2.2. Instruments

Inventory of Self-Harm Statements (ISAS); Klonsky and Glenn (2009); Spanish Version Perez et al. (2020). NSSI was assessed using the ISAS, parts I and II. Part I measured lifetime presence and frequency of 12 self-injurious behaviors. To reduce the impact of extreme values, frequency was recoded into ordinal categories: 0 (no lifetime NSSI), 1 (1–4 episodes), 2 (5–50), 3 (51–100), and 4 (>100). This categorization was based on clinical (DSM-5-TR threshold ≥ 5 episodes; American Psychiatric Association, 2022) and methodological criteria, given the association between higher frequency, greater psychopathology, and suicide risk (Andover et al., 2012) and the advantages of five-point scales for reliability (Preston & Colman, 2000). This categorization was also intended to reduce the influence of extreme frequency values, which are common in lifetime NSSI measures and often produce highly skewed distributions. By grouping observations into clinically and empirically meaningful categories, we sought to improve the stability and interpretability of the network while preserving distinctions between different levels of NSSI severity. Furthermore, higher NSSI frequency and engagement in multiple NSSI methods have consistently been associated with greater psychopathological severity and an elevated risk of future suicidal behavior (Klonsky et al., 2013; Perez et al., 2021), suggesting that meaningful distinctions exist across different levels of NSSI severity and supporting the use of categories that capture this clinical heterogeneity.

Part II assessed NSSI functions across 13 domains (intrapersonal and interpersonal), each measured with three items (total = 39). Internal consistency was excellent for both intrapersonal (ω = 0.99) and interpersonal (ω = 0.99) scales in the present sample.

Emotion dysregulation was assessed using the Difficulties in Emotion Regulation Scale (DERS) (Gratz & Roemer, 2004; Hervás & Jódar, 2008), validated for Spanish adolescents (Gómez-Simón et al., 2014). Analyses focused on the Emotional Dyscontrol subscale, which captures difficulties in managing intense and persistent negative emotional states. Internal consistency in the present sample was high (α = 0.90).

Interpersonal distress was assessed using the Interpersonal Needs Questionnaire (INQ-15; Van Orden et al., 2008), based on the Interpersonal Theory of Suicide. The scale measures perceived burdensomeness (6 items) and thwarted belongingness (9 items) on a 7-point Likert scale, with higher scores indicating greater interpersonal distress. The Spanish version validated by Perez Rodríguez et al. (2022) was used. Internal consistency was excellent for both perceived burdensomeness (α = 0.93) and thwarted belongingness (α = 0.92) in the present sample.

Hopelessness was assessed using the Hopelessness Scale (HS; Beck et al., 1974), a 20-item true–false self-report measure of negative expectations about the future (range: 0–20). The Spanish adaptation (Aguilar et al., 1995), validated in Spanish-speaking samples (Viñas Poch et al., 2004), was used. The HS has demonstrated clinical utility as a predictor of suicide risk (Beck & Steer, 1988). Internal consistency in the present adolescent sample was good (α = 0.85).

Attachment representations were assessed using the CaMir-R (Pierrehumbert et al., 1996; Spanish adaptation: Balluerka et al., 2011), a 32-item self-report measure of internal working models of attachment. Following Lacasa and Muela (2014), four dimensions were examined: Security, Parental Interference (linked to preoccupied attachment), Self-sufficiency and Resentment toward Parents (avoidant tendencies), and Childhood Trauma (associated with insecure/disorganized patterns). Items are rated on a 5-point Likert scale.

The Spanish version has shown adequate psychometric properties in adolescent samples (Balluerka et al., 2011). In the present sample, internal consistency was good for Security (α = 0.87) and Childhood Trauma (α = 0.78). Given their lower internal consistency (Parental Interference: α = 0.61; Self-sufficiency and Resentment toward Parents: α = 0.63), these subscales were excluded from subsequent analyses.

### 2.3. Procedure

The study was conducted in accordance with ethical standards and was approved by the University Ethics Committee (approval code: UCV2015-2016-25-V.2). After approval, permission to carry out the study was obtained from the school administrations of the participating institutions. Written informed consent was provided by parents or legal guardians for minors and by participants aged 18 years or older. In addition, all adolescents gave informed assent prior to participation.

Data collection was carried out on school premises by trained members of the research team. Participants completed an online self-report questionnaire administered via SurveyMonkey. Access was provided through a QR code scanned on personal mobile devices; students under 14 years of age, for whom school regulations restricted mobile phone use, completed the questionnaire using school-provided desktop computers. The survey included measures of sociodemographic characteristics and psychopathological variables.

Participation was voluntary and anonymous. Approximately 1% of eligible students declined or discontinued participation, citing reluctance to share personal or sensitive information.

### 2.4. Statistical Analyses

Descriptive statistics and zero-order Pearson correlations were computed using IBM SPSS Statistics (Version 29.0). Network analysis was conducted using JASP (Version 0.18.3) to estimate a regularized partial correlation network examining conditional associations among attachment dimensions, emotion dysregulation, hopelessness, interpersonal needs (perceived burdensomeness and thwarted belongingness), and NSSI. To enhance interpretability and reduce redundancy, only total scores or theoretically meaningful composite indicators were included. All variables were treated as continuous.

The network was estimated using the graphical Least Absolute Shrinkage and Selection Operator with the Extended Bayesian Information Criterion (EBICglasso). Pearson correlation matrices served as input, and the tuning parameter (γ) was set to 0.50. This regularization approach shrinks small partial correlations toward zero, yielding a more parsimonious and interpretable network structure. Nodes represented observed variables, whereas edges reflected regularized partial correlations controlling for all other variables in the network. The resulting network was visualized as a weighted and signed graph, with edge thickness proportional to association strength and color indicating direction (blue = positive; red = negative).

Global network characteristics were summarized in terms of the number of nodes, non-zero edges, and overall sparsity. Node importance was evaluated using standardized centrality indices, including strength, closeness, betweenness, and expected influence. In line with current recommendations, primary interpretation focused on strength and expected influence due to their greater stability and interpretability in psychological networks. Local connectivity patterns were further examined using complementary clustering metrics, including weighted clustering coefficients (Barrat et al., 2004; Onnela et al., 2005), the small-world framework (Watts & Strogatz, 1998), and weighted transitivity (Zhang & Horvath, 2005), providing additional insight into the organization of the network.

Network accuracy and stability were assessed using nonparametric bootstrapping procedures. Confidence intervals were estimated for edge weights, and centrality stability was evaluated using the correlation stability coefficient (CS-coefficient), with values above 0.25 considered acceptable and above 0.50 preferred (Epskamp et al., 2018). Centrality indices were interpreted primarily in relative terms. Missing data were handled using pairwise deletion, following standard procedures implemented in JASP for network estimation.

## 3. Results

### 3.1. Clinical Characteristics of the Sample

A total of 19.4% of participants reported lifetime engagement in NSSI. The highest prevalence was observed at ages 15 (19.7%), 13 (18.8%), and 14 (16.5%). The most frequently reported method was hitting or banging oneself (75.1%). Detailed descriptive results are presented in Table 1. The analysis and interpretation of NSSI prevalence have been reported in a previous study (Sanz-Sendra et al., 2024).

### 3.2. Correlations

Zero-order Pearson correlations are provided in Supplementary Table S1 to facilitate comparison with the regularized partial correlation network.

Overall, zero-order correlations revealed a broad pattern of positive associations among NSSI-related variables and emotional and interpersonal vulnerability factors. The strongest correlations were observed among NSSI frequency and NSSI functions (rs = 0.91–0.97). In addition, hopelessness, perceived burdensomeness, thwarted belongingness, attachment trauma, and emotion dysregulation were all moderately to strongly interrelated (rs = 0.38–0.70). Attachment security showed moderate negative correlations across the network, including with hopelessness, perceived burdensomeness, thwarted belongingness, attachment trauma, emotion dysregulation, NSSI frequency, and NSSI functions (rs = −0.29 to −0.58).

### 3.3. Network Analysis

**Figure 1:**
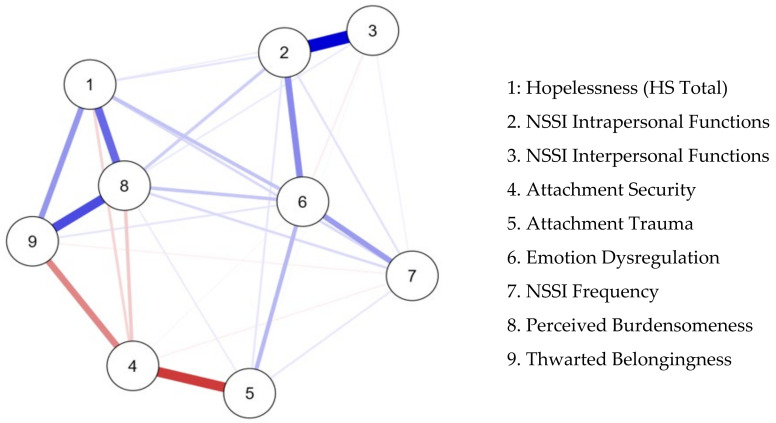
Estimated partial correlation network among study variables. Note. Blue edges represent positive associations and red edges represent negative associations. Thicker edges indicate stronger partial correlations. Nodes represent observed variables included in the network.

No redundant nodes were identified, as all variables represented theoretically distinct constructs. The estimated network comprised 9 nodes and 30 non-zero edges out of 36 possible, yielding a sparsity index of 0.17, consistent with a relatively dense network structure. Positive and negative partial correlations are depicted in Figure 1, with edge thickness proportional to association strength.

The network showed a differentiated pattern of associations. The strongest positive edge emerged between intrapersonal and interpersonal NSSI functions (0.53), followed by associations between perceived burdensomeness and thwarted belongingness (0.37), and between hopelessness and perceived burdensomeness (0.31). Emotion dysregulation was positively connected to intrapersonal NSSI functions (0.24), NSSI frequency (0.21), and attachment trauma (0.15). In contrast, attachment security showed negative associations with attachment trauma (−0.41), thwarted belongingness (−0.25), and perceived burdensomeness (−0.11), suggesting a protective pattern within the network.

**Figure 2:**
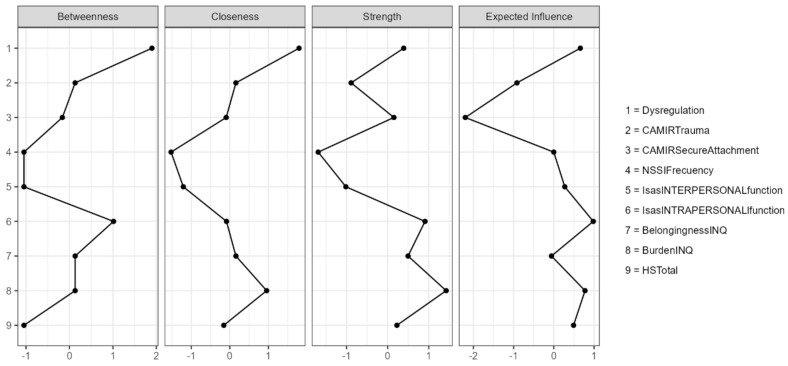
Centrality indices of the network variables. Note. The figure displays standardized centrality measures (betweenness, closeness, strength, and expected influence). Higher values indicate greater relative importance of the node within the network.

Centrality indices (Figure 2, Table 2) revealed that emotion dysregulation occupied a central position in the network, with the highest betweenness (1.90) and closeness (1.80), indicating a key role in connecting different components of the system. Intrapersonal NSSI functions also showed high centrality, with the highest expected influence (0.98) and elevated strength (0.91), supporting its relevance as a core node. Perceived burdensomeness exhibited the highest strength (1.42) and high expected influence (0.78), indicating that it is highly interconnected with other variables in the network.

In contrast, NSSI frequency and interpersonal NSSI functions displayed comparatively low or negative centrality values, with NSSI frequency showing the lowest strength (−1.69) and closeness (−1.52), suggesting a more peripheral role. Similarly, interpersonal NSSI functions showed low strength (−1.02) and closeness (−1.21). Attachment security exhibited a strongly negative expected influence (−2.20), consistent with its role as a protective factor within the network.

Clustering coefficients further characterized local connectivity patterns. Intrapersonal NSSI functions and thwarted belongingness showed relatively higher clustering values across indices, indicating stronger local interconnectedness. In contrast, attachment trauma and interpersonal NSSI functions displayed lower clustering values, suggesting weaker integration within local network neighborhoods.

Edge-weight accuracy was assessed using nonparametric bootstrapping (2000 resamples), with narrower confidence intervals observed for the strongest edges (Supplementary Figure S1), suggesting adequate accuracy of the main associations. Centrality stability was evaluated using case-dropping bootstrapping and quantified with the correlation stability coefficient (CS-coefficient). Strength demonstrated excellent stability (CS = 0.75), indicating that centrality estimates were highly robust even under substantial case removal. Visual inspection of the case-dropping bootstrap plot further supported this finding (Supplementary Figure S2), showing minimal variation in strength centrality across subsamples, even when a large proportion of cases was removed. Consequently, interpretation focused primarily on strength and expected influence, given the known limitations of other centrality indices such as closeness and betweenness (Epskamp et al., 2018).

## 4. Discussion

The aim of this study was to examine the associative structure among NSSI, emotion dysregulation, hopelessness, perceived burdensomeness, thwarted belongingness, and attachment in adolescents using a network approach. Overall, the findings suggest that NSSI is embedded within a structured system of interacting emotional and interpersonal processes, with differential contributions across nodes, challenging the assumption of homogeneous or unidirectional relationships among these variables.

The network showed a differentiated configuration depending on the centrality index considered. In terms of global influence, perceived burdensomeness and intrapersonal NSSI functions emerged as the most influential nodes, exhibiting the highest strength and expected influence. This indicates that these variables are strongly embedded within the network and may represent highly interconnected components. Their prominence suggests that these two variables occupy particularly influential positions within the estimated network: the functional meaning of non-suicidal self-injury and a key interpersonal appraisal concerning one’s perceived impact on others. Thus, the findings suggest that the network was more strongly characterized by the subjective processes that confer regulatory and interpersonal significance to NSSI than by the behavioral expression itself. This interpretation is consistent with functional models emphasizing the role of intrapersonal processes in NSSI (Klonsky, 2007; M. K. Nock, 2010).

In contrast, emotion dysregulation occupied a key structural position, with the highest betweenness and closeness values, indicating that it may function as a bridge connecting different domains of the network. Importantly, although emotion dysregulation did not emerge as one of the most central nodes in terms of global influence, its structural position suggests a distinct role in linking otherwise separate components of the system. Specifically, emotion dysregulation was linked to attachment-related experiences, interpersonal vulnerability, and NSSI-related processes within the estimated network. Its associations with intrapersonal NSSI functions, NSSI frequency, and attachment trauma reinforce this interpretation. Rather than being associated with a single domain, emotion dysregulation was connected to multiple areas of the network, suggesting a potentially important position within the overall configuration of associations, articulating affective vulnerability, functional meaning, and relational adversity. This is consistent with previous evidence identifying emotion dysregulation as a central transdiagnostic process in NSSI (Wolff et al., 2019).

Peripheral nodes included NSSI frequency and interpersonal NSSI functions, which showed comparatively low or negative centrality values. This pattern suggests that these variables may reflect more distal or outcome-like components rather than core mechanisms within the network. Notably, although NSSI frequency is often interpreted as an indicator of severity (De Luca et al., 2023), the present findings indicate that it is less central than intrapersonal functions. Thus, the network was more strongly characterized by the psychological and regulatory meaning of the behavior rather than its frequency. Similarly, interpersonal NSSI functions, despite their association with intrapersonal processes, occupied a relatively peripheral position, suggesting that the network is more strongly structured by intrapersonal regulation than by the social functions of NSSI.

The interpersonal domain nevertheless displayed a coherent and structurally meaningful configuration. Perceived burdensomeness and thwarted belongingness were strongly interconnected and jointly linked to hopelessness, a pattern consistent with the Interpersonal Theory of Suicide (Joiner, 2005; Van Orden et al., 2010). Within this subsystem, perceived burdensomeness exhibited greater centrality, suggesting a particularly prominent structural role, according to recent evidence (Perez Rodríguez et al., 2025). Hopelessness, in contrast, occupied an intermediate position and was primarily connected to interpersonal variables. Taken together, these findings support the presence of a coherent interpersonal risk cluster rather than isolated associations, underscoring the structural relevance of interpersonal vulnerability within the network, consistent with recent network studies indicating strong interconnections and bridging roles of interpersonal factors in NSSI (He et al., 2025).

Attachment findings further reinforce this systemic interpretation. Attachment security exhibited a strongly negative expected influence and negative associations with key risk variables, supporting its role as a protective factor within the network. In contrast, attachment trauma was positively linked to emotion dysregulation, suggesting that adverse attachment-related experiences may be incorporated into the network through regulatory difficulties. These findings are consistent with attachment theory and empirical evidence linking insecure attachment to emotion dysregulation and increased vulnerability to NSSI (Gander et al., 2021; Pallini et al., 2020). From this perspective, attachment was associated with several key variables in the network and may represent an important relational context for understanding these associations within the ongoing configuration of emotional and interpersonal processes. This interpretation is also in line with Shaver and Mikulincer (2007), who propose that, under stress, individuals automatically activate internal representations of attachment figures, thereby enhancing perceived security. Accordingly, NSSI may be understood as embedded within a broader system of vulnerability shaped by attachment representations.

Clustering analyses further characterized local network organization. Intrapersonal NSSI functions and thwarted belongingness showed higher clustering, indicating stronger local interconnectedness, whereas attachment trauma and interpersonal NSSI functions were less locally integrated. This suggests that some nodes are not only globally central but also embedded within tightly connected local structures (Borsboom & Cramer, 2013; Watts & Strogatz, 1998). In particular, the local cohesion of intrapersonal NSSI functions further supports their role as a core component of the system (He et al., 2025; Robinaugh et al., 2016), whereas the weaker local integration of attachment trauma and interpersonal functions is consistent with their more peripheral structural position (Boschloo et al., 2015; Hepp et al., 2020; Taylor et al., 2018).

From a methodological perspective, these findings underscore the added value of network analysis. Whereas zero-order correlations suggested a diffuse pattern in which variables appeared broadly interconnected, the partial correlation network revealed a differentiated structure of relationships. This distinction highlights the limitations of linear approaches and supports the conceptualization of psychopathology as a system of interacting components rather than a simple chain of independent effects (Borsboom & Cramer, 2013).

Although centrality metrics provide useful information about the relative position and connectivity of nodes within a network, recent methodological work has questioned the extent to which centrality can be interpreted as evidence of causal influence, clinical relevance, or optimal intervention targets, particularly in cross-sectional networks (Bringmann et al., 2019). Highly central nodes may not necessarily exert greater causal effects on other nodes, nor does their identification imply that targeting these variables will lead to broader changes throughout the network. Therefore, the present findings should be interpreted cautiously and regarded as hypothesis-generating. Further longitudinal and experimental studies are needed to determine whether the central nodes identified in the present network play a causal role in the maintenance of NSSI and whether they represent effective targets for intervention.

If replicated in longitudinal research, these findings may have implications for interventions targeting emotion regulation, the functional meaning of NSSI, and maladaptive interpersonal cognitions, particularly perceived burdensomeness in adolescents. This perspective is consistent with treatments focusing on regulatory processes, such as dialectical behavior therapy (Linehan, 1993; Linehan et al., 2006), as well as with functional models conceptualizing NSSI as a strategy for emotion regulation and interpersonal communication (M. Nock, 2009). Furthermore, it aligns with the interpersonal theory of suicide, which highlights perceived burdensomeness as a key risk factor (Joiner, 2005; Van Orden et al., 2010), and with attachment-informed approaches aimed at strengthening relational security (Cassidy et al., 2018; Mikulincer & Shaver, 2016).

Several limitations should be acknowledged. First, the cross-sectional design precludes causal inference. Accordingly, the network should be interpreted as reflecting patterns of conditional associations rather than causal or temporal relationships among variables. Future longitudinal studies are needed to clarify the directionality and temporal dynamics of the observed associations. In addition, all variables were assessed through self-report, which may introduce shared method variance. Moreover, two theoretically relevant attachment dimensions (Parental Interference and Self-sufficiency and Resentment toward Parents) were excluded due to their relatively low internal consistency in the present sample. Although this decision was intended to reduce measurement error in network estimation, it may have influenced the resulting network structure. Future research should examine whether similar patterns emerge when these attachment dimensions can be assessed with greater reliability. Furthermore, although the sample size was large, participants were recruited through a convenience sampling procedure, and nearly two-thirds of the sample came from a single autonomous community. Therefore, the findings should be generalized to the broader Spanish adolescent population with caution. In addition, certain analytical decisions, including the categorization of NSSI frequency, may have reduced variability and resulted in some loss of information. However, this approach was considered preferable to the inclusion of highly skewed raw frequency counts that could disproportionately influence network estimation. These limitations warrant cautious interpretation of the findings.

Despite these limitations, the present study provides an integrative perspective by examining, within a single network, domains that are typically studied in isolation. Overall, the findings suggest a differentiated pattern of associations in which perceived burdensomeness and intrapersonal NSSI functions occupied particularly central positions, emotion dysregulation occupied a highly connected position linking multiple domains, interpersonal variables were closely interconnected, attachment was associated with several key variables, and NSSI frequency occupied a more peripheral position. Future longitudinal research is needed to clarify the temporal dynamics and directionality of these associations.

## Figures and Tables

**Table 1:** Descriptive characteristics of the sample.

Variable	Category	*n*	%
NSSI		401	19.4
Frequency (lifetime episodes)	0–4	6	0.3
	5–50	81	3.9
	51–100	42	2.0
	≥101	272	13.2
Age (years)	12	60	15.0
	13	75	18.8
	14	66	16.5
	15	79	19.7
	16	56	14.0
	17	48	12.0
	18	16	4.0
Methods	Hitting or banging oneself	301	75.1
	Severe scratching	300	74.8
	Biting	283	70.6
	Pinching	277	69.1
	Wound picking	24	62.1
	Hair pulling	218	54.4
	Rubbing against rough surfaces	169	39.9
	Cutting	146	36.4
	Needle sticking	71	17.7
	Ingesting chemicals	58	14.5
Functions	Affect regulation	372	92.8
	Anti-dissociation	335	83.5
	Self-punishment	335	83.5
	Marking distress	305	76.1
	Self-care	304	75.8
	Toughness	284	70.8
	Anti-suicide	252	62.8
	Interpersonal boundaries	248	61.8
	Interpersonal influence	170	42.4
	Peer bonding	106	26.4

Note. Percentages for frequency are calculated based on the total sample, whereas percentages for methods, age and functions are calculated among participants reporting NSSI.

**Table 2:** Centrality measures of the estimated network (*N* = 2068).

Number of Nodes = 9	Number of Non-Zero Edges = 30/36		Sparsity = 0.167	
Variable	Betweenness	Closeness	Strength	Expected Influence
Hopelessness (HS Total)	−1.047	−0.156	0.225	0.490
NSSI Intrapersonal Functions	1.014	−0.087	0.909	0.981
NSSI Interpersonal Functions	−1.047	−1.206	−1.016	0.272
Attachment Security	−0.164	−0.094	0.152	−2.201
Attachment Trauma	0.131	0.158	−0.889	−0.917
Emotion Dysregulation	1.898	1.795	0.390	0.659
NSSI Frequency	−1.047	−1.523	−1.689	−0.001
Perceived Burdensomeness	0.131	0.955	1.423	0.776
Thwarted Belongingness	0.131	0.158	0.496	−0.059

Note. Values represent standardized centrality indices. Higher values indicate greater relative importance of the node within the network. Betweenness reflects the extent to which a node acts as a bridge between other nodes; closeness indicates the average distance of a node to all other nodes; strength represents the sum of absolute edge weights connected to a node; expected influence accounts for both the strength and direction (positive or negative) of associations.

## Data Availability

The datasets generated during and/or analysed during the current study are available from “NSSI and attachment in Spanish adolescents”, https://doi.org/10.7910/DVN/YVHAXK, Harvard Dataverse, V1, UNF:6:IJnpXJbmx78DU16t1bqypw== [fileUNF].

## Supplementary Materials

The following supporting information can be downloaded at: https://www.mdpi.com/article/10.3390/ejihpe16070088/s1, Figure S1: Bootstrap Edge-Weight Accuracy Plot of the Estimated Network; Figure S2: Case-dropping bootstrap analysis of strength centrality stability. Table S1: Descriptive statistics and zero-order Pearson correlations among study variables (*N* = 2067).
