# Supplementary material for: Mapping the Network Structure of Non-Suicidal Self-Injury: The Role of Emotional and Interpersonal Vulnerability and Attachment in Spanish Adolescents
Source: Eur J Investig Health Psychol Educ. 2026 Jun 25;16(7):88. doi: 10.3390/ejihpe16070088 (PMC13408751; doi:10.3390/ejihpe16070088)
Supplement: Supplementary file 1 [file ejihpe-16-00088-s001.zip › ejihpe-4374769-supplementary.pdf]

Table S1

Descriptive statistics and zero-order Pearson correlations among study variables (N = 2067)

Variable	1	2	3	4	5	6	7	8	9
1. Hopelessness	—								
2. Perceived Burdensomeness	.67***	—							
3. Thwarted Belongingness	.62***	.70***	—						
4. NSSI Intrapersonal Functions	.37***	.39***	.28***	—					
5. NSSI Interpersonal Functions	.35***	.37***	.28***	.97***	—				
6. NSSI Frequency	.35***	.37***	.28***	.92***	.91***	—			
7. Attachment Security	−.50***	−.55***	−.58***	−.30***	−.29***	−.29***	—		
8. Attachment Trauma	.38***	.42***	.39***	.30***	.28***	.28***	−.58***	—	
9. Emotion Dysregulation	.50***	.52***	.46***	.46***	.43***	.42***	−.41***	.42***	—

Note. Values represent Pearson correlation coefficients. All correlations were significant at $p < .001$.

Missing data were handled using pairwise deletion.

Figure S1.

Bootstrap Edge-Weight Accuracy Plot of the Estimated Network.

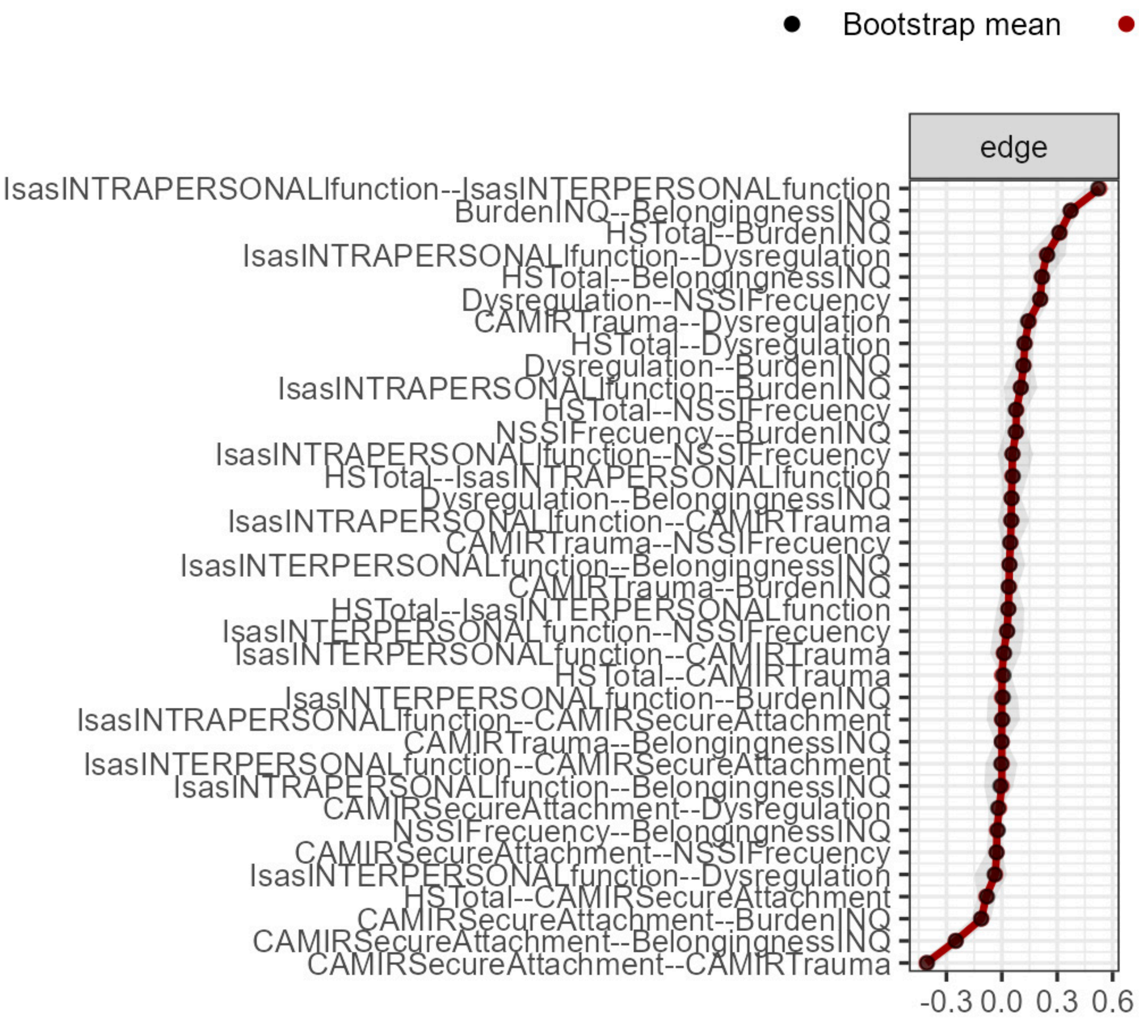

Note: Bootstrapped confidence intervals of edge weights. Each line represents an edge in the network, with points indicating the estimated edge weight and surrounding intervals reflecting bootstrapped variability. Narrower intervals indicate greater accuracy of edge estimation.

Figure S2.

Case-dropping bootstrap analysis of strength centrality stability.

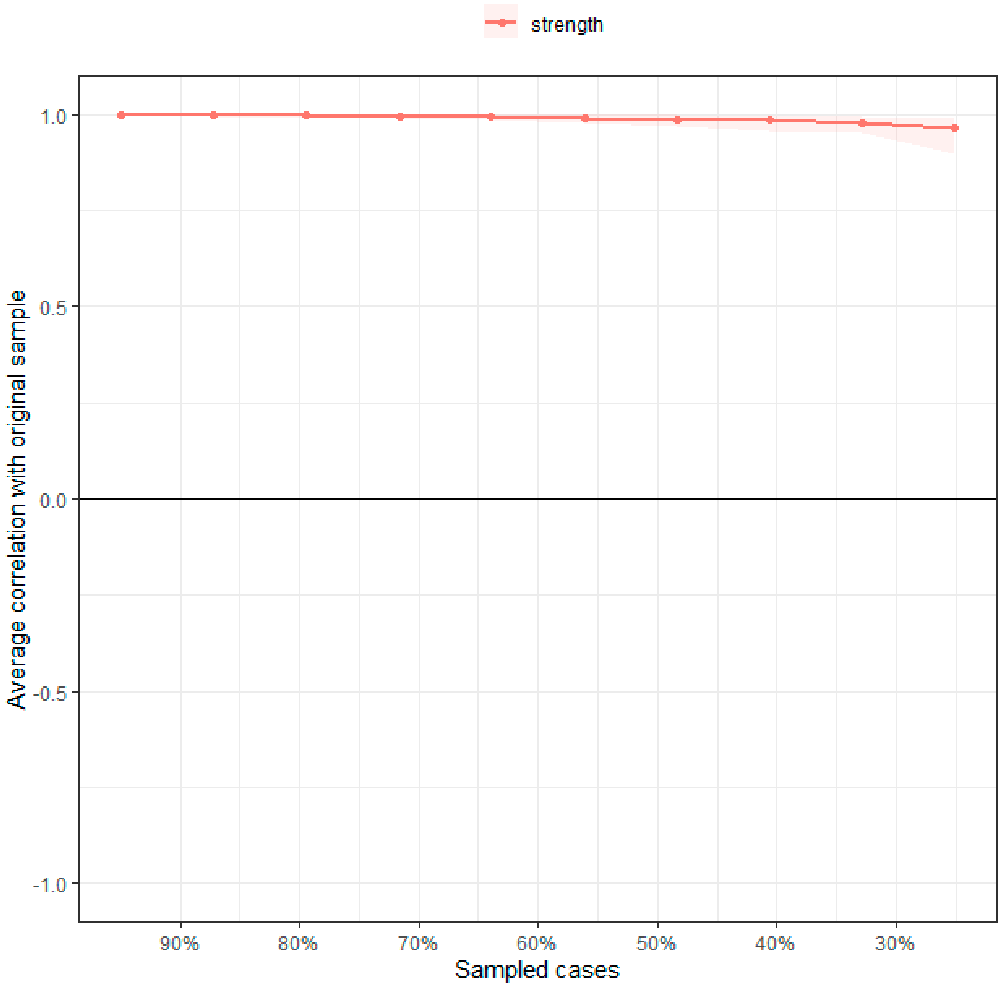

Note: The plot displays the average correlation between strength centrality indices estimated in subsamples and those obtained in the original network as a function of the proportion of cases retained. The relatively flat curve and high correlations across all sampling levels indicate excellent stability of strength centrality, even under substantial case removal.
